# Ethical and regulatory aspects of facial scanning in dentistry: a scoping review

**DOI:** 10.1007/s00784-026-07132-y

**Published:** 2026-09-22

**Authors:** Thanatchaporn Jindanil, Pierre Lahoud, Maxime Ducret, Raphael Richert, Jennifer K. Wagner, Reinhilde Jacobs

**Affiliations:** 1 OMFS-IMPATH Research Group, Department of Imaging and Pathology, KU Leuven, Campus St. Rafael, Kapucijnenvoer 7, 3000 Leuven, Belgium; 2 Department of Oral and Maxillofacial Surgery, University Hospitals Leuven, Leuven, Belgium; 3 Department of Radiology, Chulalongkorn University, Bangkok, Thailand; 4 Department of Periodontology and Oral Microbiology, Department of Oral Health Sciences, KU Leuven, Leuven, Belgium; 5 Department of Conservative Dentistry, Periodontology and Digital Dentistry, LMU University Hospital, LMU Munich, Munich, Germany; 6 Hospices Civils de Lyon, PAM d’Odontologie, Lyon, France; 7 Faculty of Odontology, Lyon 1 University, Lyon, France; 8 Laboratoire de Biologie Tissulaire Et Ingénierie Thérapeutique, UMR5305, CNRS/UCBL, Lyon, France; 9 LaMCoS, UMR5259, INSA Lyon, CNRS, Villeurbanne, France; 10 School of Engineering Design and Innovation, Penn State University, University Park, Pennsylvania United States of America; 11 Department of Anthropology, Penn State University, University Park, PA United States of America; 12 Department of Biomedical Engineering, Penn State University, University Park, PA United States of America; 13 Institute for Computational and Data Sciences, Penn State University, University Park, PA United States of America; 14 Huck Institutes of the Life Sciences, Penn State University, University Park, PA United States of America; 15 Rock Ethics Institute, Penn State University, University Park, PA United States of America; 16 Penn State Dickinson Law, University Park, PA United States of America; 17 Department of Dental Medicine, Karolinska Institutet, Alfred Nobels Allé 8, 141 04 Huddinge, Sweden

**Keywords:** Facial scan, Facial-driven, Digital dentistry, Digital health, Data governance

## Abstract

**Objectives:**

To provide an overview of facial scanners and scanning applications used in dentistry, focusing on clinical validation, certification status, and to identify ethical and regulatory concerns.

**Materials and methods:**

A literature search was performed with eligibility criteria that included studies in dentistry that used at least one facial scanner or smartphone scanning application in human facial region in any language. Scanners and applications identified from literature and International Dental Show 2025 were manually noted, and subsequently searched for detailed certification (medical, general, others) and technical information. Furthermore, studies explicitly/implicitly mention/discuss ethical and/or regulatory concerns were included.

**Results:**

Ninety-five scanners and 23 smartphone-based applications were identified, of which 90 scanners and all applications were clinically validated. Six scanners (6%, FusionFaceScanner, Carestream CS9600, Planmeca ProFace, Planmeca ProMax 3D Mid, Planmeca ProMax 3D ProFace, Planmeca Viso G7) were Medical Device Regulation-certified, 49% held other certifications, and 51% had unverifiable certification status. Among 19,789 records, six scientific studies discussed ethical concerns, including ethical approval, informed consent, privacy, accuracy, and medical regulatory obligations.

**Conclusion:**

Despite validation, publicly accessible, verifiable medical-related certification remained limited among the identified scanners, highlighting that ethical and regulatory considerations are underreported in the scientific literature rather than absent from clinical practice. Informed consent, data privacy, accuracy, and medical device compliance were addressed in the included studies.

**Clinical relevance:**

As facial scans provide comprehensive information for esthetic- and facial-driven treatment planning, the findings of this study highlight the need for practitioners and researchers to remain cautious regarding device safety, regulatory compliance, and technological limitations.

**Supplementary Information:**

The online version contains supplementary material available at https://doi.org/10.1007/s00784-026-07132-y.

## Introduction

The popularity of facial scanning has tremendously increased with the rise of digital dentistry, as it provides a more comprehensive tool for diagnosis, treatment planning, and outcome evaluation in many oral and maxillofacial applications [1]. This approach enables clinicians to obtain accurate digital representations of patients’ facial features and their relationship to dental structures. Even though facial photographs have long been used clinically, the trend toward facial integration shifts from two-dimensional (2D) to three-dimensional (3D) imaging, driven by technological advancements, providing more information for quantitative morphological assessments and comprehensive communication [2, 3].

The facial scan is the most critical component of a 3D facial model, serving as the primary visual unit for both patients and clinicians. Over the years, the underlying scanning technologies have substantially evolved, from early laser triangulation systems to photogrammetry, stereophotogrammetry, and structured-light scanners [4, 5]. These advancements have aimed to shorten acquisition time and minimize scanning artifacts [6]. Despite these improvements, each technology still has inherent limitations that users must carefully consider, and the cost of commercial scanners continues to limit their accessibility in the day-to-day practice. More recently, the introduction of smartphones equipped with high-resolution cameras, TrueDepth sensors, and Light Detection and Ranging (LiDAR), combined with dedicated scanning applications, has provided a practical alternative for clinical use [7–9].

**Fig. 1:**
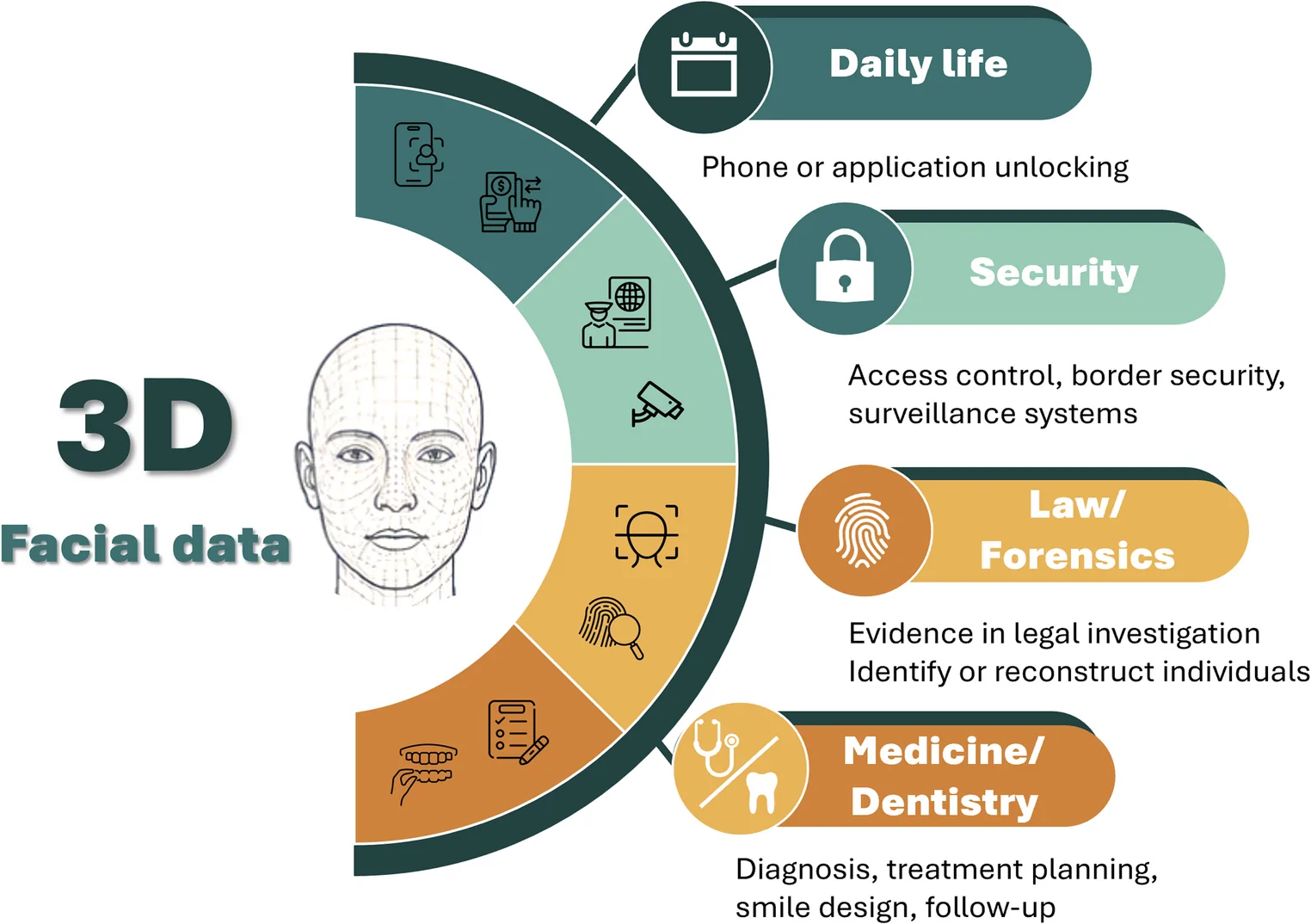
Overview of the applications of three-dimensional facial data

Although facial scanning and facial recognition both rely on capturing facial information and therefore share similar concerns about the non-anonymized nature of the face, their ethical and legal implications differ considerably. Facial recognition technology, which identifies individuals based on their facial features, raises significant ethical, legal, and social concerns [10–12]. In contrast, although the use of facial scans in dentistry is increasing, the scale and associated risks of implementation may be considered comparatively small compared with those of the aforementioned counterpart (Fig. 1). Although principles such as diversity, transparency, wellness, privacy protection, prudence, law and governance, and sustainability were recognized as essential for AI-integrated smile design [13], there remains a notable lack of clear, consolidated guidance on the ethical, legal, and practical considerations specific to facial scan data in clinical and research contexts. This gap is especially prominent concerning regulatory frameworks and formal certifications, such as FDA (Food and Drug Administration) clearance in the United States and CE (Conformité Européenne) marking in Europe.

Thus, this study aimed to provide an overview of facial scanners and scanning applications, with a particular focus on their clinical validation, certification status, and to explore concerns and awareness regarding the ethical and legal considerations associated with the use of facial scans in dentistry. The main focus was on addressing this critical translational gap, rather than proposing a new paradigm of biometric ethics. The hypothesis was that a substantial proportion of facial scanners and scanning applications used in dentistry were clinically validated and medical-certified, and there were no ethical or regulatory concerns.

## Materials and methods

To address the aforementioned questions, this review was organized into two interconnected parts. Part 1 provided an overview of facial scanners and scanning applications identified in the literature alongside the scanners debuted at the International Dental Show (IDS) 2025 in Cologne, which is the premier global showcase for dental state-of-the-art. It is the industry’s primary venue for launching the newest and transformative technologies and debuting significant breakthroughs to the global market. The focus of this part was on technical specifications and certification data retrieved from the secondary search of the company or manufacturer databases. Part 2 consisted of a scoping review examining concerns and awareness regarding the ethical and legal considerations of facial scanning in articles.

### Part 1: Technical specification and certification of the scanners identified from the literature review

#### Protocol and registration

The protocol for the scoping review was prepared in accordance with the PRISMA Extension for Scoping Reviews (PRISMA-ScR), available at http://prisma-statement.org. The framework was created in line with the EQUATOR Network’s recommendations for developing high-quality, transparent reporting guidelines in health research. In addition, the study protocol was pre-registered on the Open Science Framework (https://doi.org/10.17605/OSF.IO/67JXZ).

#### Eligibility criteria

The inclusion criteria for this section covered all facial scanners, a purpose-built device specifically to capture high-resolution 3D images, or a smartphone scanning application, a software-based mobile device to generate a 3D model used in the facial region, as identified during the study selection process- reports assessed for eligibility. The sample of facial scanners identified from the literature review was augmented by the inclusion of facial scanners that had been showcased at the IDS in Cologne, Germany, in March 2025.

#### Information sources

To identify relevant publications, the following electronic databases were searched for studies published up to December 2025 on Pubmed, Ovid, Cochrane, Embase, Scopus, IEEE Xplore, ACM library, clinicaltrials.gov, ICTRP (WHO), and preprints (bioRxiv, medRxiv). The initial search strings, mainly focused on facial scan and facial scanner, were developed by two reviewers (TJ and PL) and refined through discussions with all authors. The finalized search strategies, along with the database-specific adaptations, are presented in Supplementary Table 1. All search results were then exported into EndNote reference manager (version 20, Clarivate Analytics, Pennsylvania, USA) for duplicate removal. A further reference check through the reference lists of key articles was conducted to identify additional relevant articles.

To obtain technical specifications and audited marketing material related to accreditation details, search engines were used to locate official company websites and product pages for each scanner, as well as relevant user manuals. The retrieved websites were documented on the web archive. Furthermore, emails were sent to companies and manufacturers with available contact information to request additional information (physical characteristics (weight, portability), optical configurations (lighting, receptors, wavelength), spatial capacity (scan range, volume), performance (speed, accuracy, resolution), and additional details ( software, certification) and verification of the collected data. Technical specifications for smartphone scanning applications were sourced directly from the official Apple AppStore and Google PlayStore descriptions. The approach relied on manufacturer-reported and publicly available materials, rather than verified regulatory status.

#### Data collection and results synthesis

One reviewer (TJ) identified the scanners and scanning applications during the article selection process, and the selection was subsequently verified by a second reviewer (PL). For data extraction, both reviewers designed two charts. The first chart captured information sourced from official company websites and user manuals, including: company details (name and country), scanner or application name, type of scientific validation (internal validation included technical validation: tested on mannequin or model, external validation included technical report: tested on a human subject, and clinical validation: tested on multiple human subjects), launch year, and current availability on the market. Accreditation status for commercial scanners (FDA general or medical, CE general or medical, and other certifications) was noted, while user personal information collection for smartphone applications was obtained. The applications collected personal information, including contact details, user identifiers, diagnostics, location, usage data, and user content. These were recorded as yes, no, or unverifiable, and, when applicable, the exact types of data collected were categorized as identifiable or unidentifiable and specified.

The second chart focused on technical specifications. For all scanners, data were tabulated on alignment method, weight (Light: < 3 kg, Mid: 3–10 kg, Heavy: > 10 kg), capture speed (Fast: < 1 s (s), > 30 frames per second (fps), > 1,000,000 points per second (pps), Moderate: 1–5 s, 10–29 fps, 500,000–1,000,000 pps, Compromised: > 5 s, < 10 fps, > 500,000 pps), accuracy (High: < 0.1 mm, Standard: 0.1–1 mm, General: > 1 mm), resolution (Basic: VGA, 1 MP, 8-bit, Professional: Full-HD, 1–5 MP, 24-bit, Advanced: > 5 MP, 24-bit), and availability of in-house software. Additional information was collected based on specific technologies: laser class and wavelength for laser scanners, portability, lighting, and number of reference points for photogrammetry, stereophotogrammetry, and structured-light systems, and underlying technology, camera used (front or rear), latest software update, version, languages available, and application categories (Medical, graphic and design, photo and video, utilities) for smartphone applications. To organize and synthesize the data, the first reviewer (TJ) created an initial Excel sheet, which was refined through discussion with the second reviewer (PL) and then converted into a Word table for the final version.

### Part 2: Scoping review of the literature

#### Eligibility criteria

The inclusion criteria were relevant studies in the dental field that used a facial scanner or a smartphone scanning application in the human facial region for clinical or scientific purposes, with explicit (direct and clear arguments) or implicit (inferred through the use of related terms or concepts) mentions and/or discussions on ethics and regulations. Exclusion criteria included studies that did not use facial scanners or smartphone applications in their methods, used a model or mannequin head, involved other parts of the body, were conference abstracts, or were systematic reviews.

#### Study selection

Two reviewers (TJ and PL) first conducted an initial calibration exercise by screening the same set of articles’ titles and abstracts to ensure consistency in the review process. Full-text assessments were subsequently conducted by both reviewers to determine eligibility according to the inclusion criteria. Any disagreements were resolved through discussion or, when necessary, by consulting a senior advisor (RJ).

#### Data charting process, data extraction, and result synthesis

The Excel data-charting sheet was initially prepared by the first reviewer (TJ), subsequently rechecked by the second reviewer (PL). The following information were collected from each included article: article characteristics (authors, year of publication), study characteristics (field of use, type, aim, and scanning technology employed e.g. laser, stereophotogrammetry, structured light, smartphone application), and ethical (ethical approval, informed consent, privacy) and legal (technical accuracy, regulatory consideration, others) concerns mentioned and/or discussed. ChatGPT (version 5.2) was used solely to enhance manuscript readability, with full authors’ oversight.

## Results

### Part 1: Technical specification and certification of the scanners identified from the literature review

**Fig. 2:**
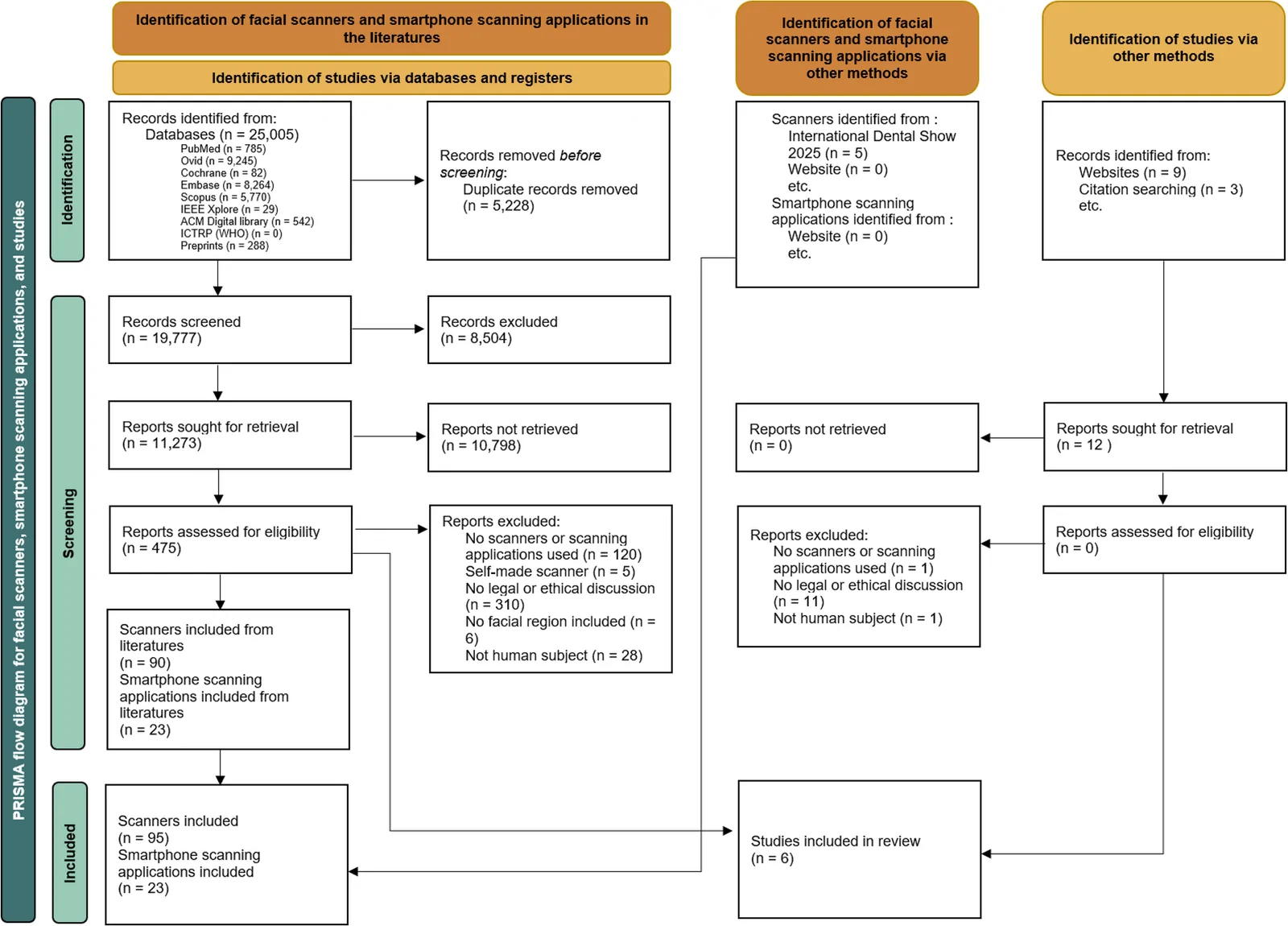
Flow diagram for facial scanners, scanning applications documentation, and article selection

**Fig. 3:**
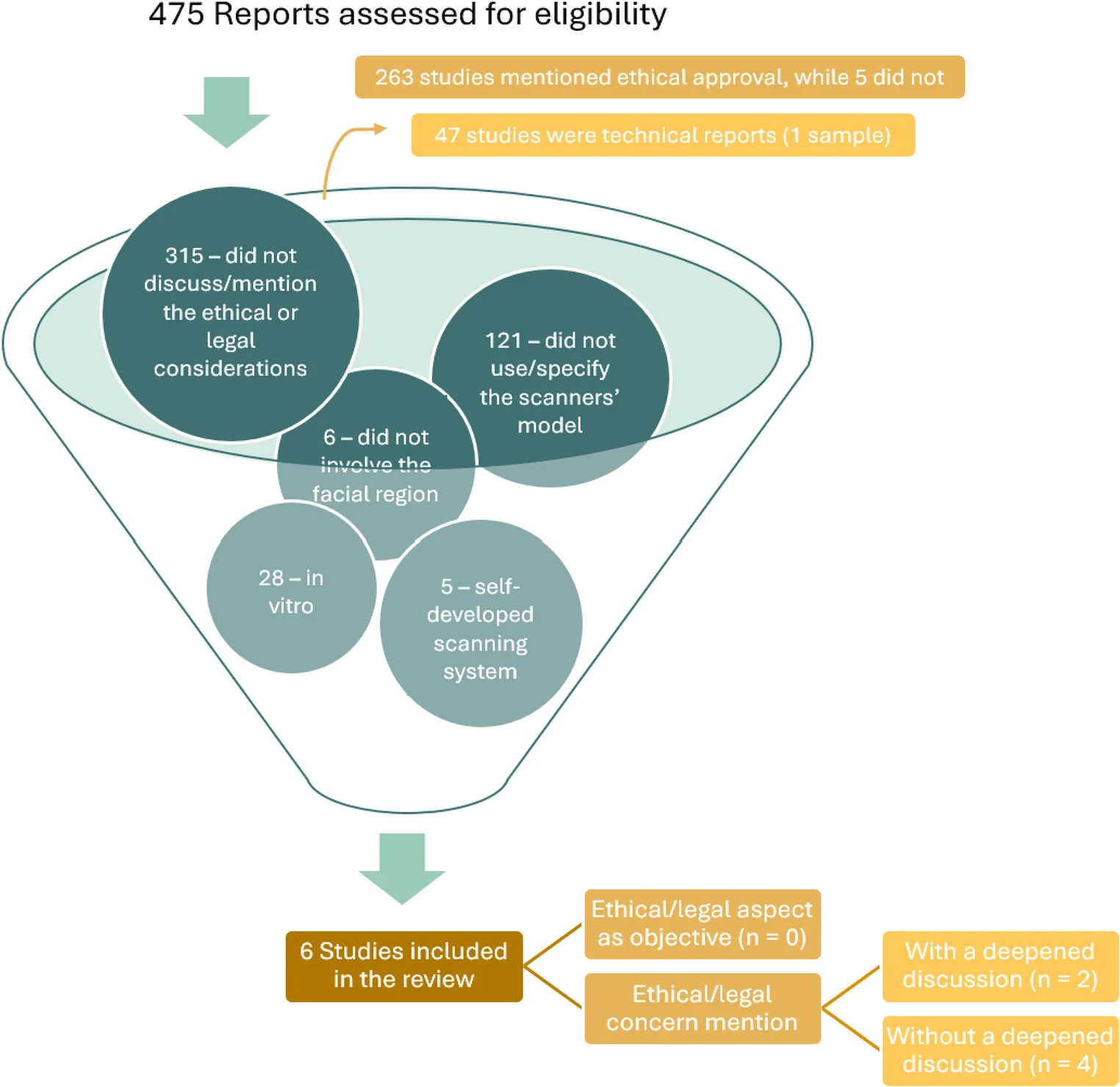
Overview of the study selection process for part 2: scoping review of the literature, showing the number of reports assessed for eligibility, screened, assessed for inclusion, and included in the final analysis

Based on the inclusion criteria, 90 scanners and 23 smartphone scanning applications were noted during the article screening process from 475 eligible articles since 2000. Five additional scanners were added from the IDS (JMFace (3DIFY, Shenzhen, China), T2000 (Fussen, Shenzhen, China), Imager S100 (Hansfive, Hangzhou, China), FusionFaceScanner (LargeV, Beijing, China), and Fanyu 3D (Scedent, Shanghai, China)). The selection process is shown in the flow diagram in Fig. 2 (dark brown column) and Fig. 3. Of 95 scanners noted, 11 were laser scanners (12%), 27 were based on photogrammetry or stereophotogrammetry (28%), and 57 were structured light scanners (60%).

Table 1 presents scientific validation and certification/accreditation status of the facial scanners used in dentistry. A total of 52 companies across 12 countries were identified as developers of commercial scanners, with the highest number from China (28%), followed by the United States (24%), Germany (12%), Japan, Luxembourg, South Korea, and the United Kingdom (each 6%). France and Italy each accounted for 4%, while Brazil and Finland each accounted for 2%. In addition, 17 developers from six countries were identified for smartphone scanning applications, with the United States as the headquarters for the majority (71%).

**Table 1:**
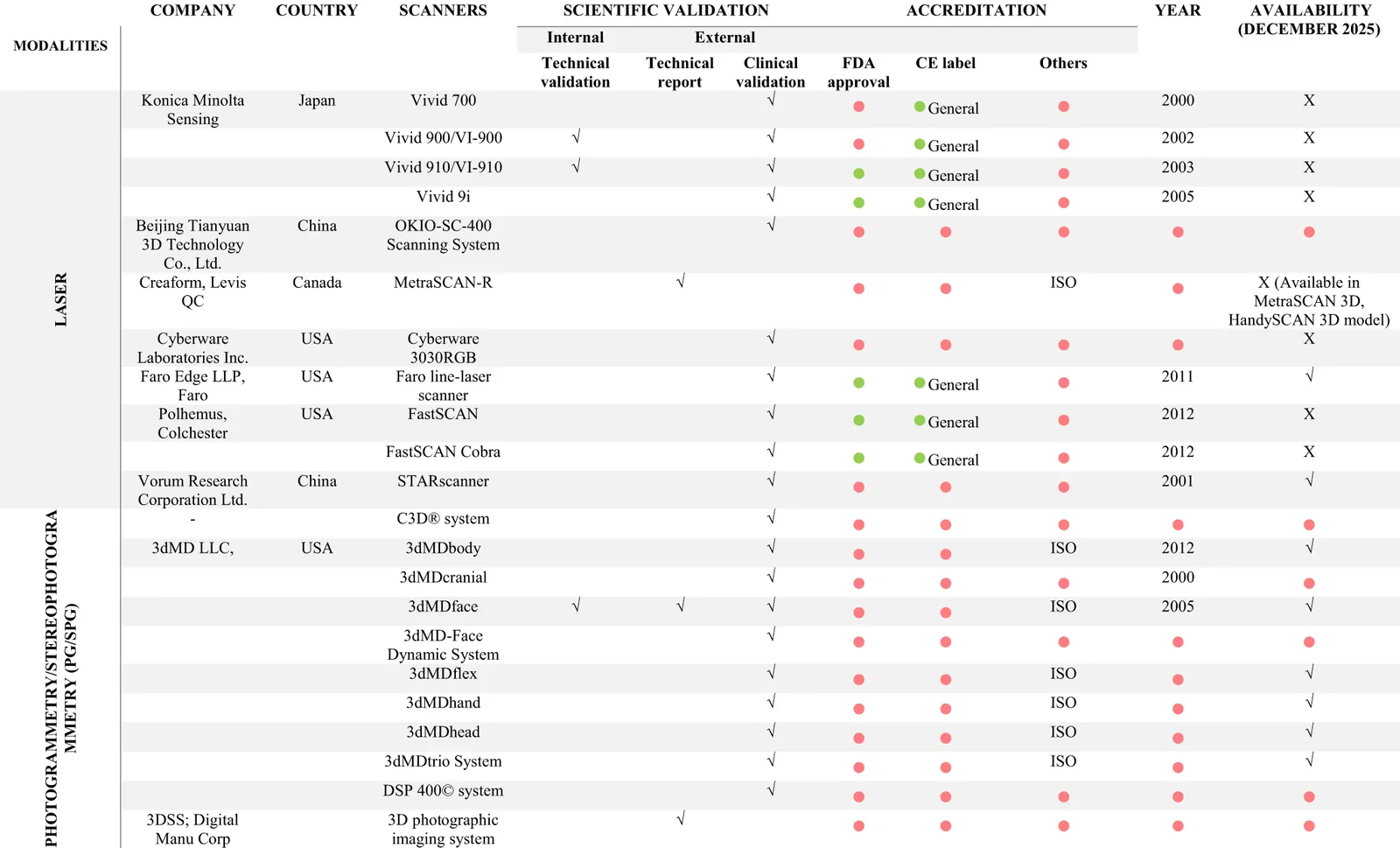

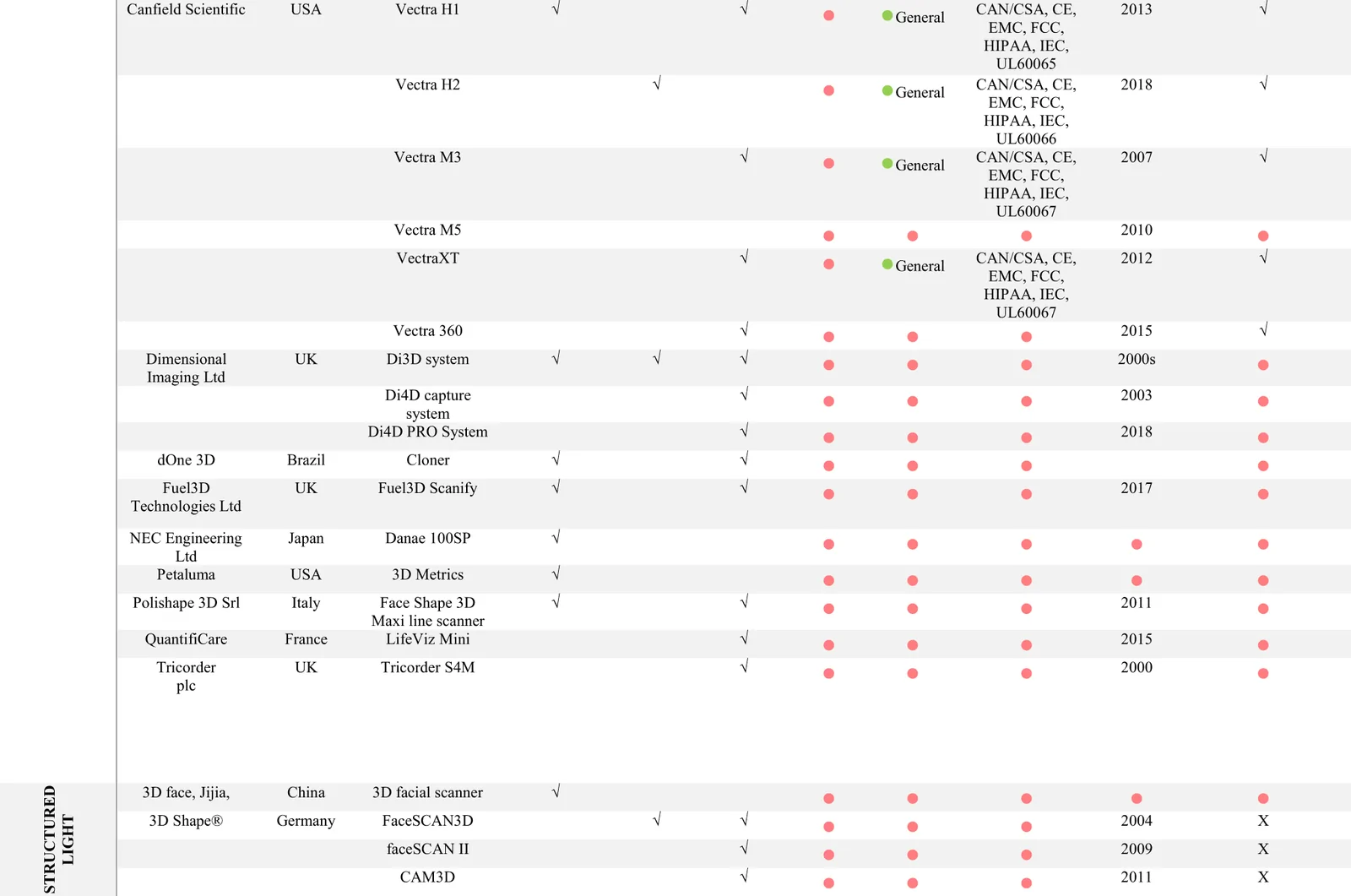

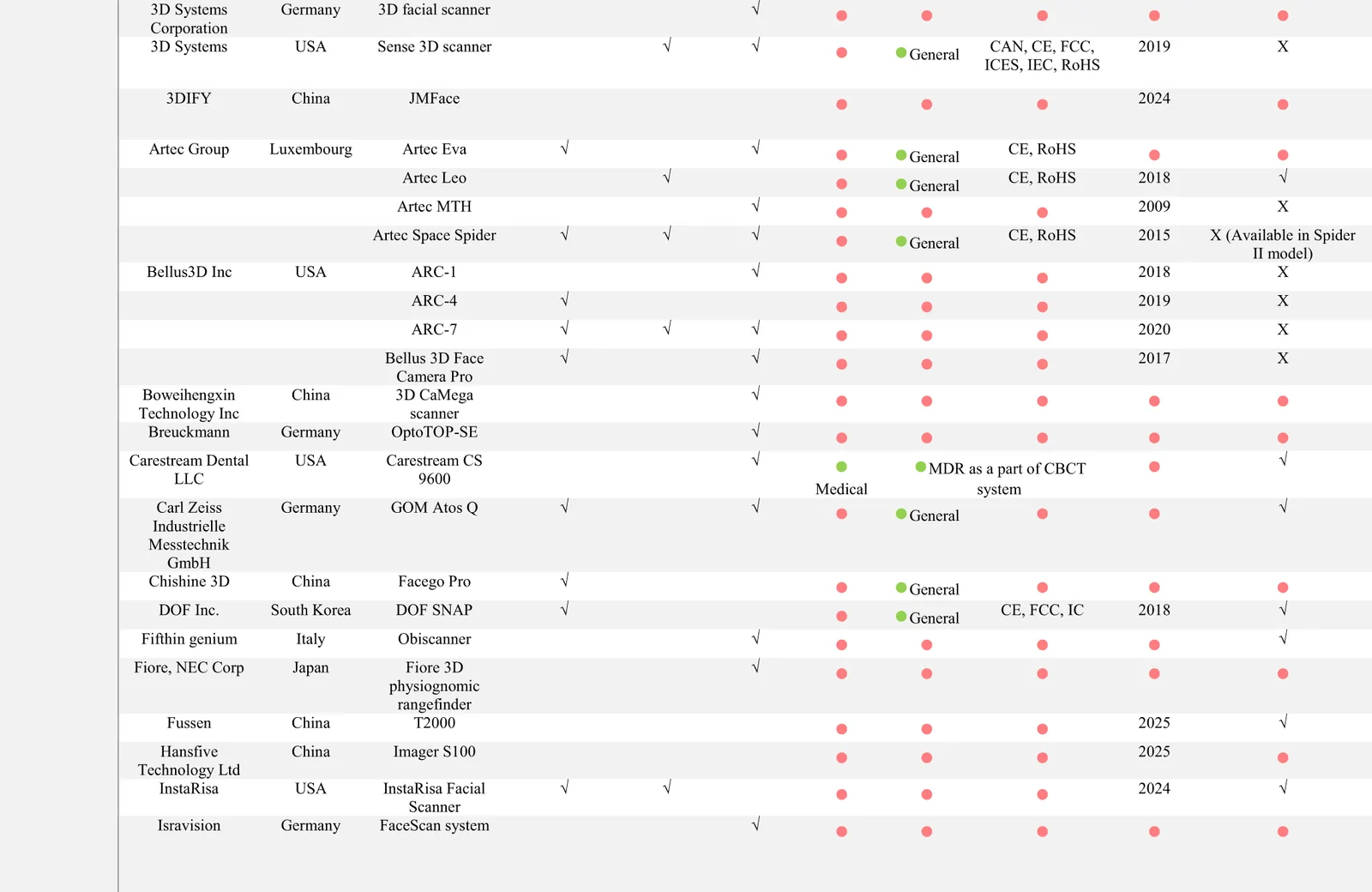

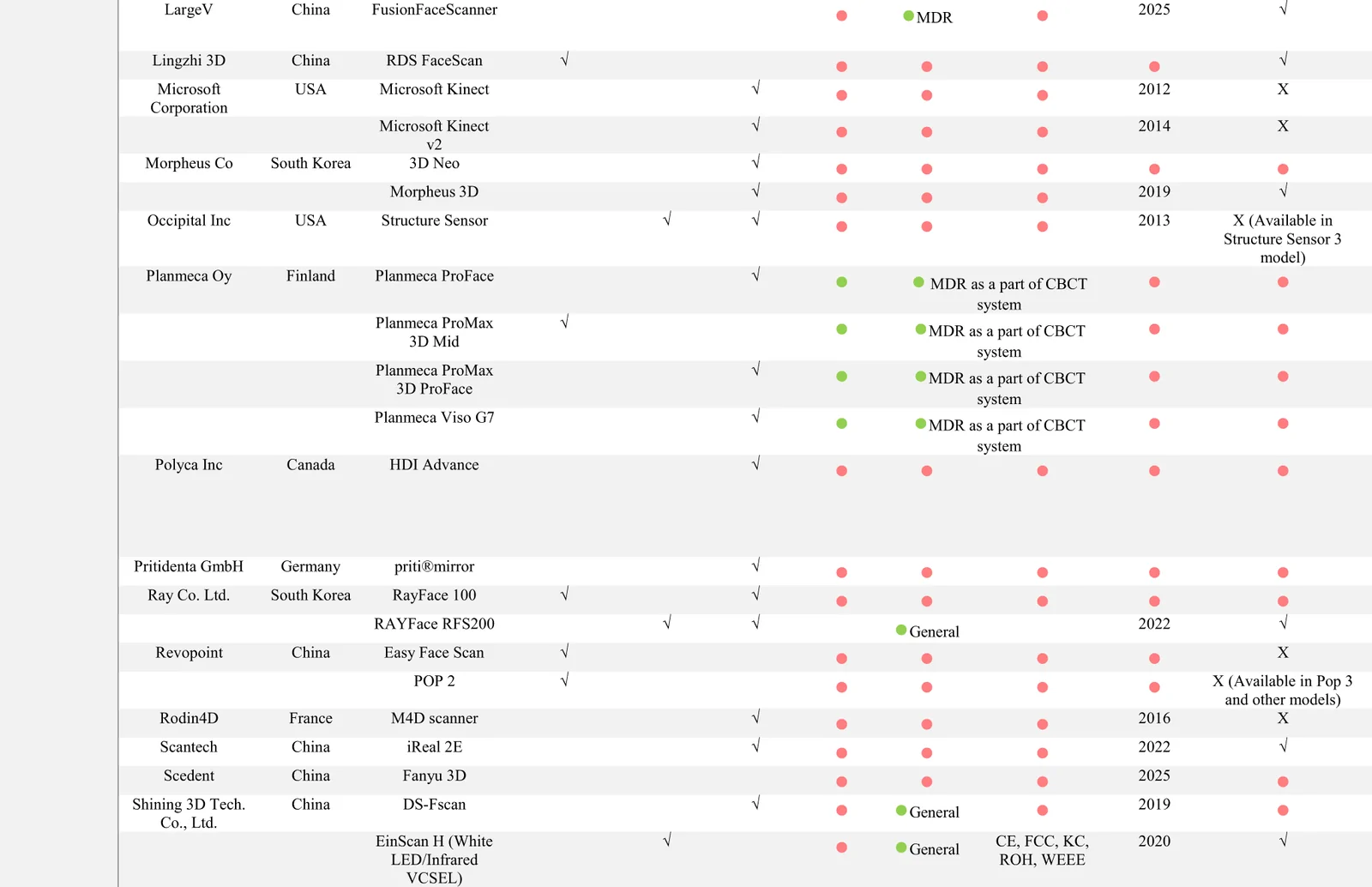

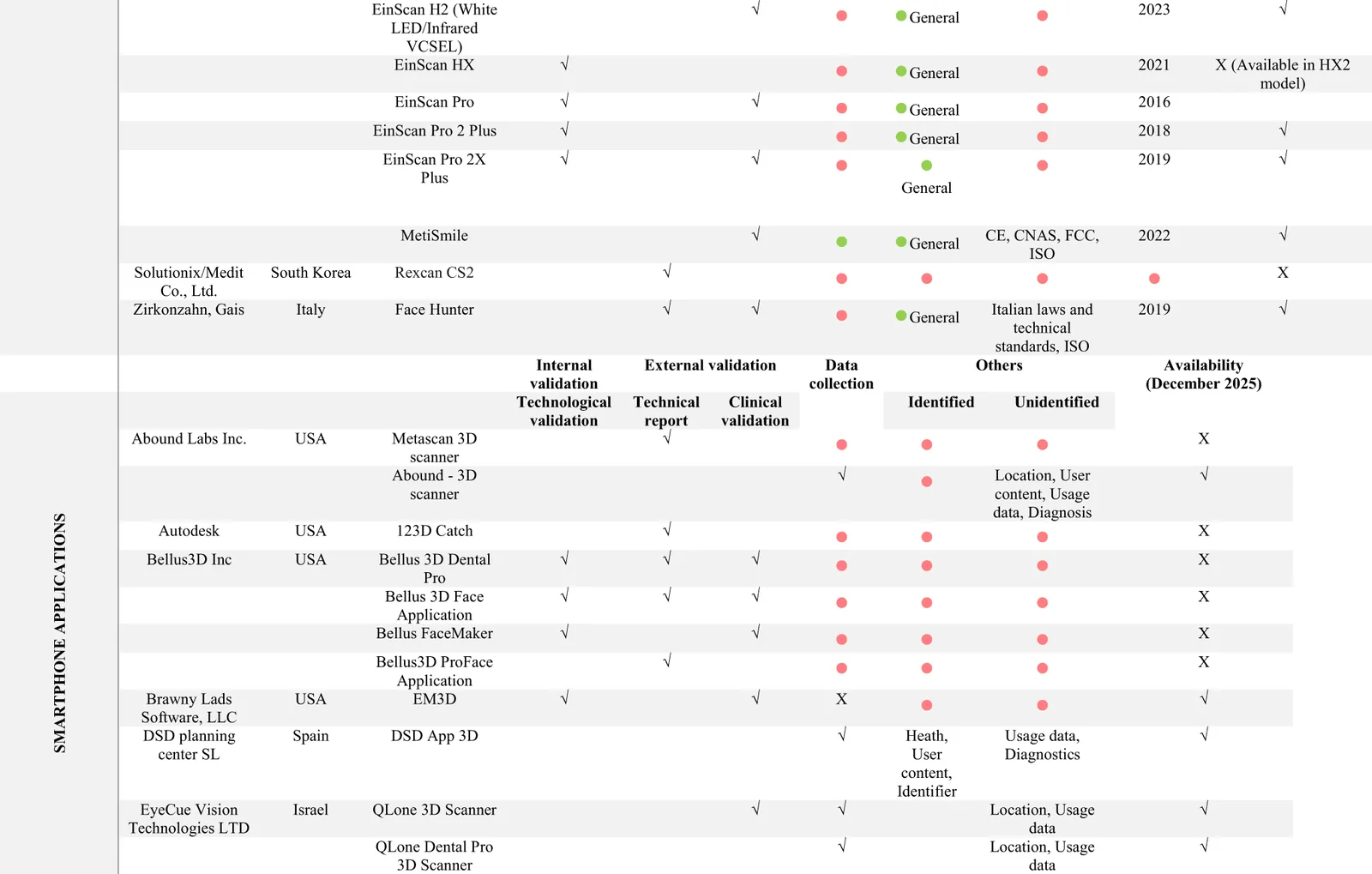

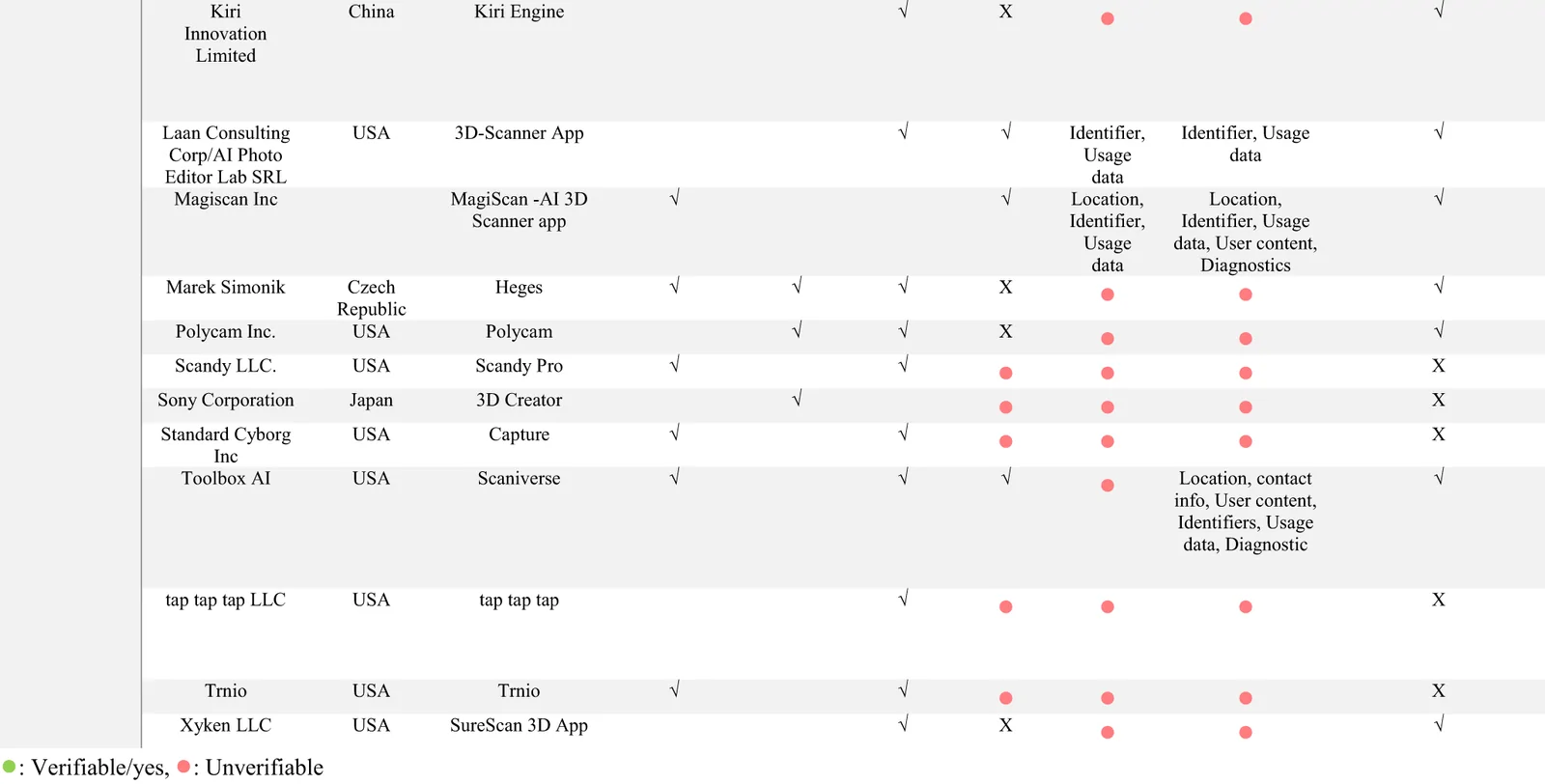
Scientific validation and certification/accreditation of facial scanners and scanning applications

Regarding the scientific validation of scanners and smartphone scanning applications for the facial region, the highest proportion of scientifically validated devices was laser scanners (91%), followed by photogrammetry and stereophotogrammetry systems (89%), structured-light scanners (82%), and smartphone applications (60%).

For accreditation, structured light scanners integrated with cone beam computed tomography (CBCT) systems received medical device accreditation as part of the CBCT unit. Furthermore, the static structured light FusionFaceScanner (LargeV, Beijing, China), launched in 2025, was explicitly stated to be an MDR-certified. This represented 10% of all structured light scanners and 6% of the total facial scanners reviewed. Other forms of accreditation, such as general FDA approval, CE marking for products marketed in the European Union, and standard certifications including ISO standards or national regulatory compliance, were reported for 73% of laser scanners, 37% of photogrammetry and stereophotogrammetry systems, and 40% of structured-light scanners. Notably, accreditation status remained unverifiable for approximately 51% of the scanners identified in the literature, indicating a substantial gap in regulatory clarification, as the compliance data is publicly inaccessible to users.

In smartphone scanning applications, 30% reported collecting user data, and within this group, 43% collected identifiable information, including user content, personal identifiers, health-related data, usage data, and location data. In contrast, 22% of the applications explicitly stated that they do not collect patient data; of these, five applications were still available as of December 2025 (EM3D (Brawny Lads Software, LLC, USA), Kiri Engine (Kiri Innovation Limited, China), Heges (Marek Simonik, Czech Republic), Polycam (Polycam Inc, USA), and SureScan 3D App (Xyken LLC, USA)).

**Fig. 4:**
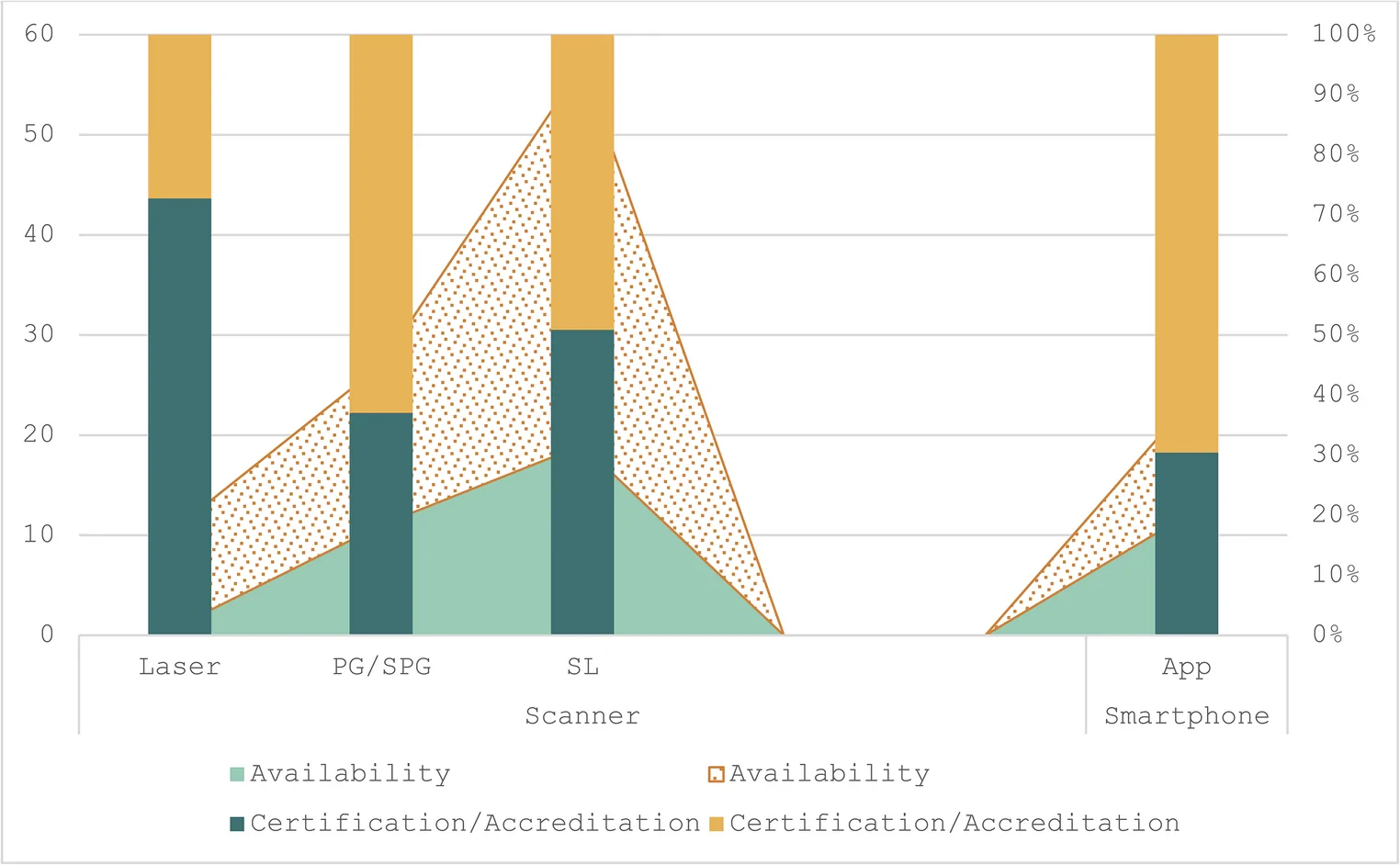
Overall availability and certification/accreditation status of facial scanners by scanning technologies, and data collection practices of smartphone scanning applications in December 2025

Among the scanners identified in the literature and at the IDS, as of December 2025, structured light scanners were the most currently available (*n* = 19), followed by smartphone-based applications (*n* = 12), photogrammetry and stereophotogrammetry systems (*n* = 11), and a laser scanner. This total excluded discontinued models that have been replaced by newer versions (one stereophotogrammetry and four structured light). Data for the remaining scanners (*n* = 59, 53%) was unavailable. An overall certification/accreditation obtained and availability are illustrated in Fig. 4.

Table 2 shows the technical specifications of facial scanners and smartphone scanning applications. Laser scanners, introduced in the early 2000s, demonstrated high accuracy, but the speed ranged from compromised to fast, with mostly mid-sized weights, and they mostly adhered to defined laser safety classifications. Photogrammetry and stereophotogrammetry systems offered mostly moderate-to-fast capture time and were available in static, portable, and dynamic configurations with lightweight. Structured light scanners also enabled rapid acquisition and provided a broad range of portability. The currently available commercial scanners can provide either professional or advanced resolution. Meanwhile, smartphone scanning applications based on TrueDepth, LiDAR, or photogrammetry technologies offered the lowest-cost alternative. However, the update timing varied, and only the DSD App 3D (DSD Planning Center SL, Spain) was clearly classified in medical category.

**Table 2:**
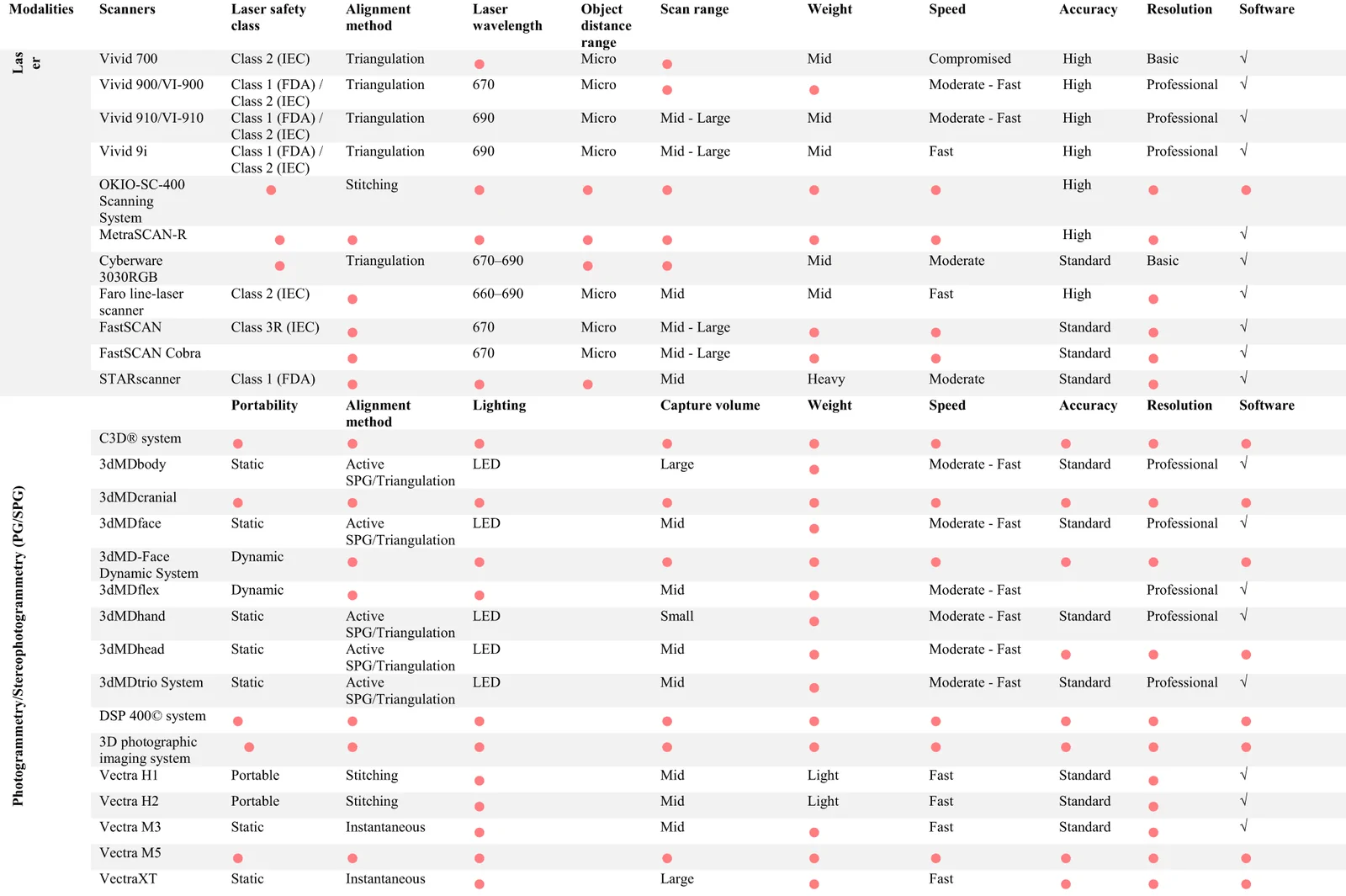

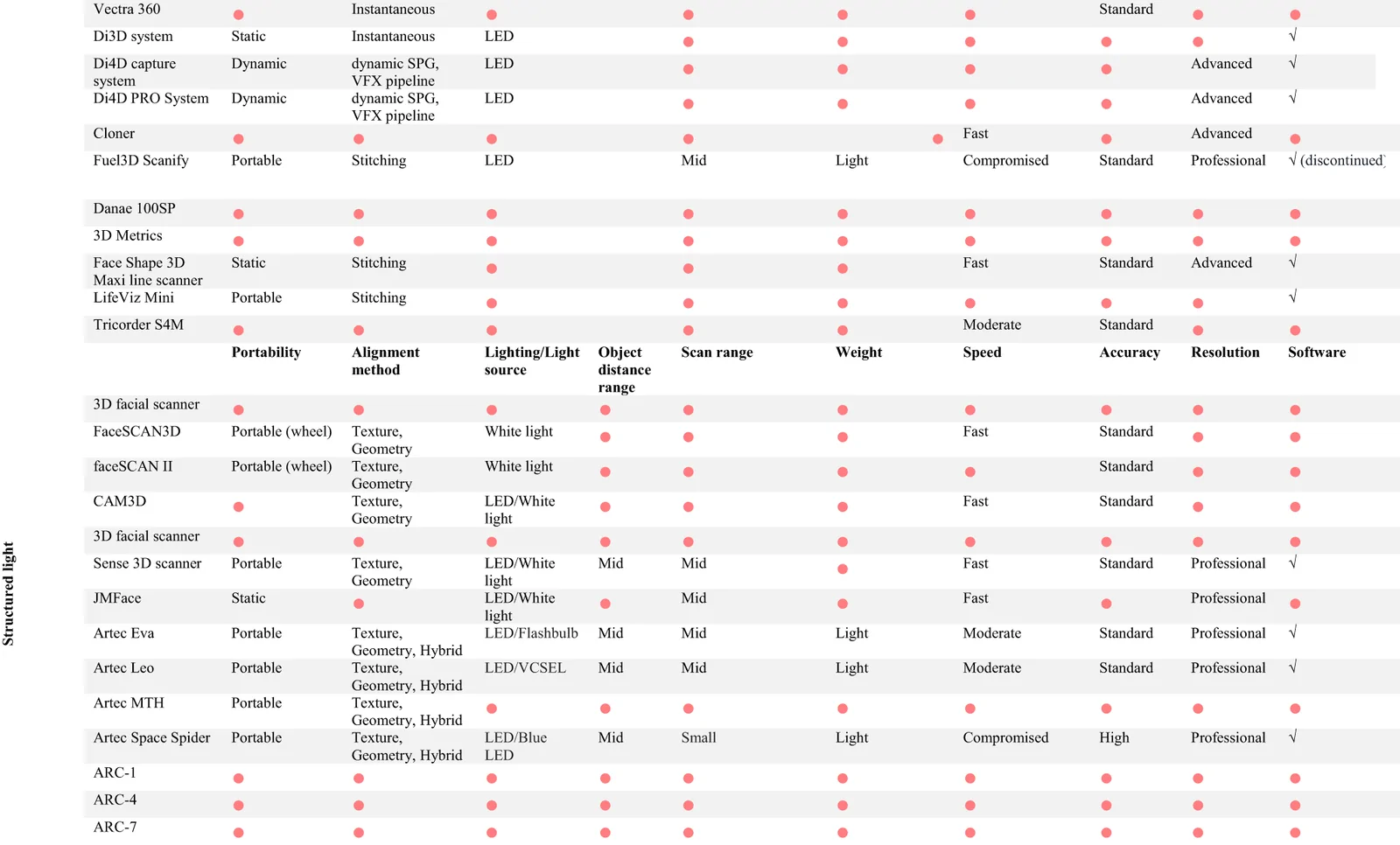

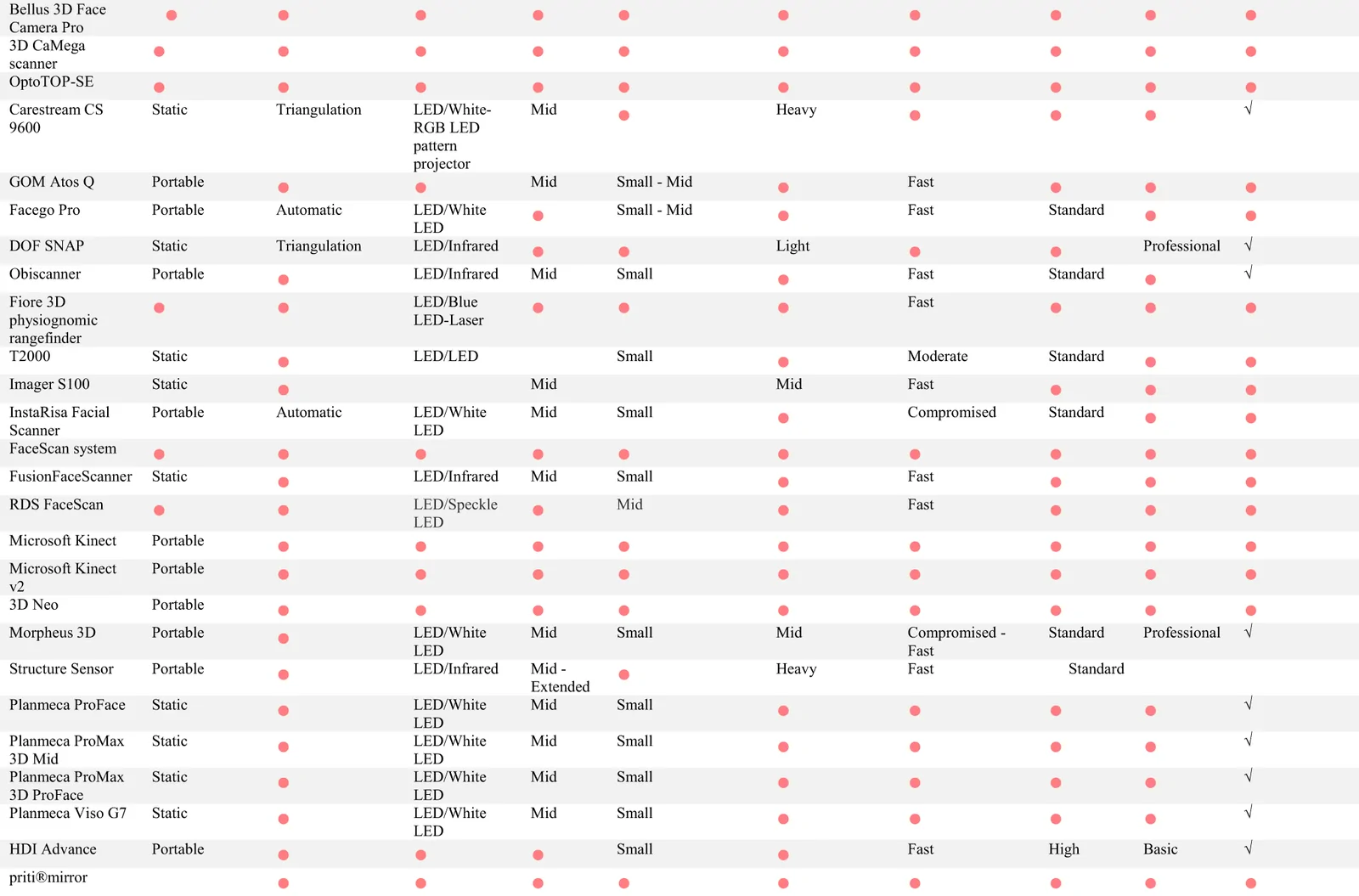

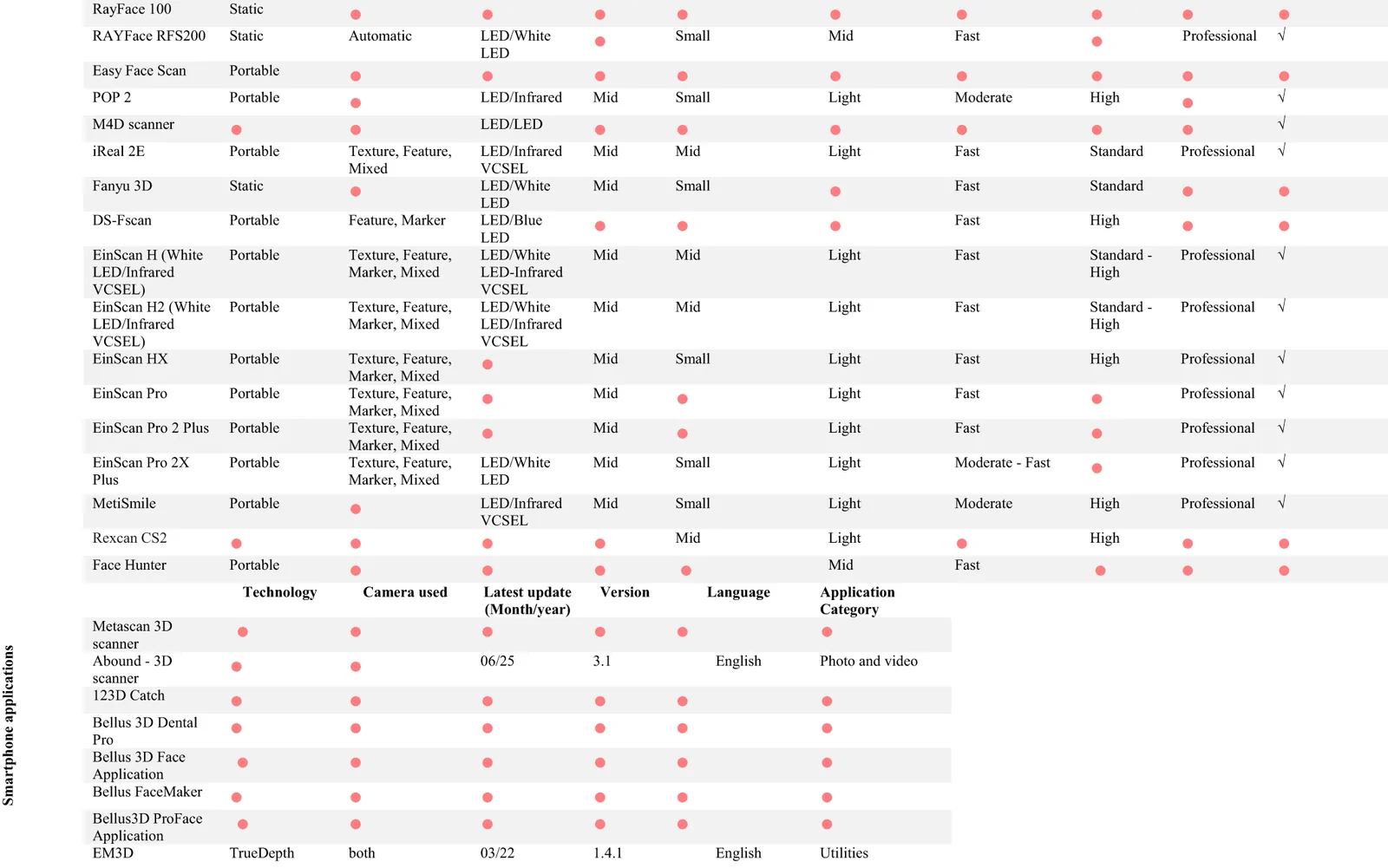

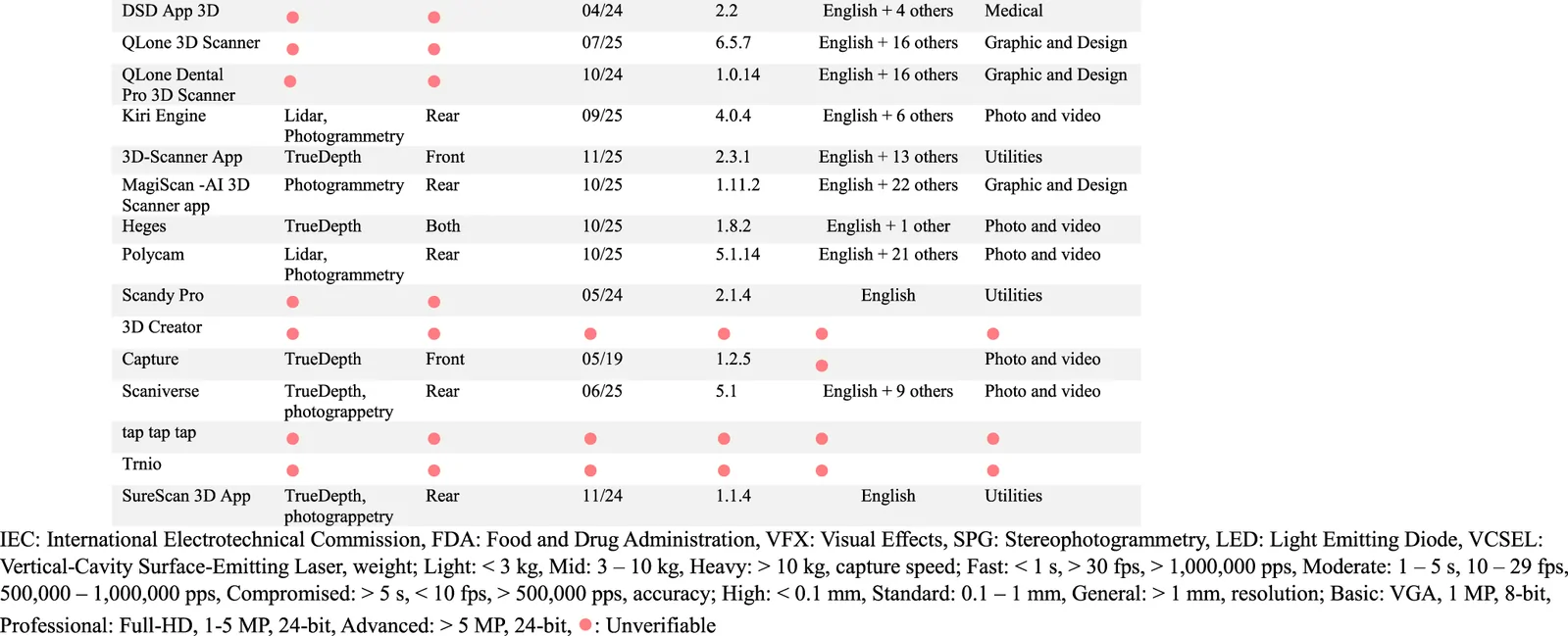
Technical specification of facial scanners and scanning applications

IEC: International ElectrotechnicalCommission, FDA: Food and Drug Administration, VFX: Visual Effects, SPG:Stereophotogrammetry, LED: Light Emitting Diode, VCSEL: Vertical-CavitySurface-Emitting Laser, weight; Light: < 3 kg, Mid: 3 – 10 kg, Heavy: >10 kg, capture speed; Fast: < 1 s, > 30 fps, > 1,000,000 pps,Moderate: 1 – 5 s, 10 – 29 fps, 500,000 – 1,000,000 pps, Compromised: > 5 s,< 10 fps, > 500,000 pps, accuracy; High: < 0.1 mm, Standard: 0.1 – 1mm, General: > 1 mm, resolution; Basic: VGA, 1 MP, 8-bit, Professional:Full-HD, 1-5 MP, 24-bit, Advanced: > 5 MP, 24-bit, ●: Unverifiable

**Fig. 5:**
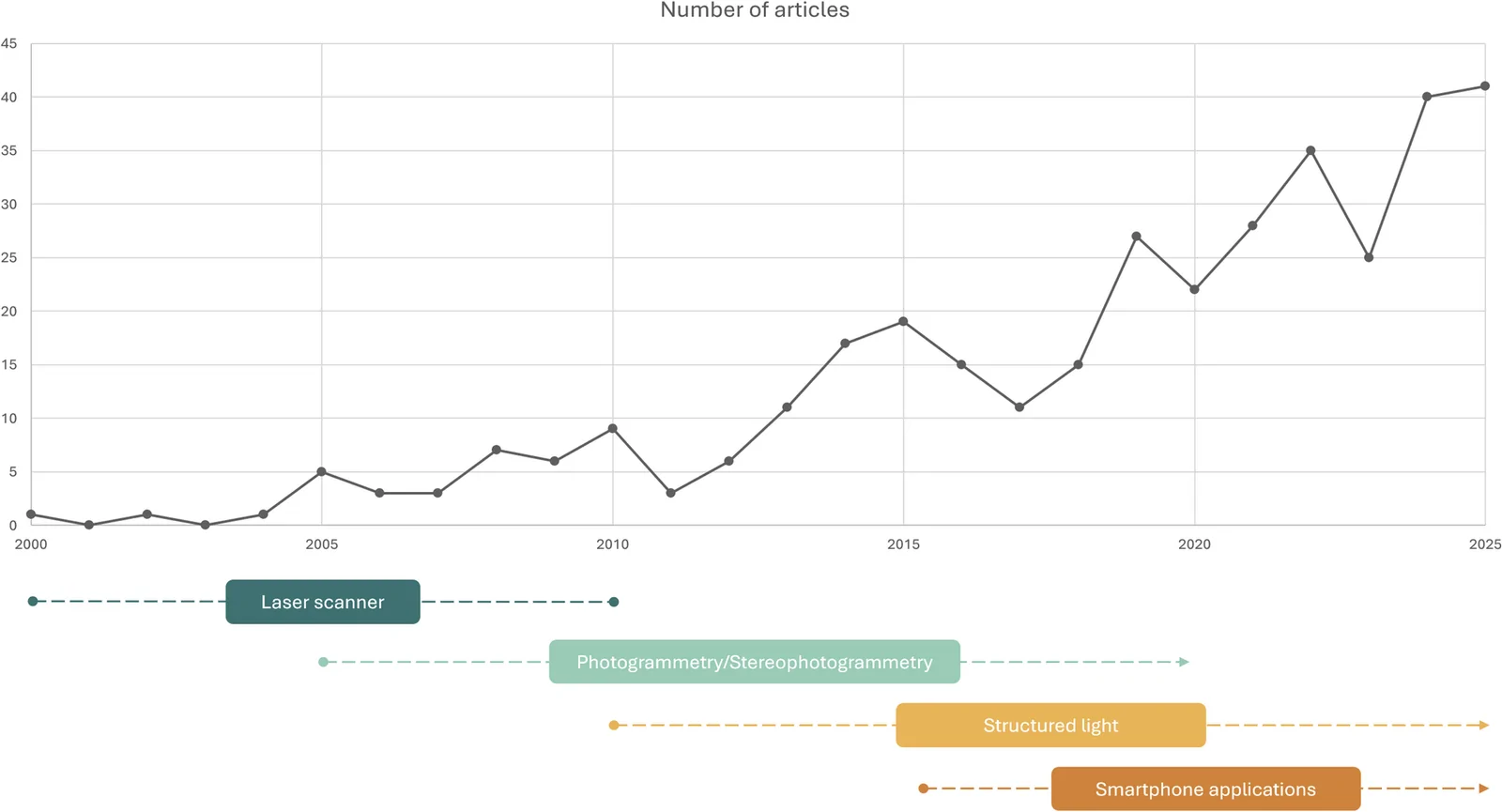
Publications trending with the use of facial scanners or scanning applications

Figure 5 presents a comprehensive overview of publication trends for facial scanners and smartphone-based scanning technology.

### Part 2: Scoping review of the literature

Following duplicate removal, 19,789 records were retrieved from electronic databases, and six studies [14–19] met the inclusion criteria. The selection process is shown in the flow diagram in Fig. 2 (light brown column).

The details of the included studies are presented in Table 3. The included studies were from the field of dentistry (50%), including general dentistry (33%) [15, 16], oral and maxillofacial surgery (17%) [13], and forensics (50%) [16–18]. All included studies were experimental in design and used facial scans obtained through laser scanners (17%) [18], stereophotogrammetry (50%) [16–18], structured light systems (50%) [14–16], or smartphone-based scanning applications (33%) [15, 16].

**Table 3:** Data extracted from the selected studies

Author	Year	Field	type	Aim	Scanning technology	Concern/awareness						
						Ethical				Legal		
						Ethical approval	Informed consent	Privacy	Accuracy		Regulatory consideration	Others
Jindanil, et al	2024	Dentistry(General)	Experimental	To quantitatively and qualitatively assess five 3D facial scanners with the soft tissue outline on CBCT as a reference	Portable SL		√	√	√			System validation
Jindanil, et al	2025	Dentistry(General)	Experimental	To quantitatively and qualitatively assess five 3D facial scanners with the soft tissue outline on CBCT as a reference	Static SPG, portable SPG, portable SL, smartphone apps		√	√ (GDPR)			√ (Medical device regulation)	Purpose limitation, transparency
Kaul, et al	2025	Dentistry(Surgery)	Experimental	To assess the accuracy and feasibility of the EM3D smartphone application with the handheld structured light scanner for aural data acquisition	Portable SL, smartphone app	√					√ (Medical regulatory consideration	
Koudelová, et al	2015	Forensics	Experimental	To confirm facial age changes at the age of 12-15, evaluate facial aging trajectories, propose and test age progression model	Portable SPG		√		√			Accuracy for legal implementation
Koudelová, et al	2019	Forensics	Experimental	To construct aging trajectory from 7-17 and propose three-dimensional age progression	Portable SPG		√		√			Accuracy for legal implementation
Lynnerup, et al	2009	Forensics	Experimental	To compare the cranial measures obtained by direct measurement, skull craniometric landmark, and surface scanner	Laser		√		√			Accuracy for surveillance

*SL s*tructured light, *SPG* stereophotogrammetry, *App *Application, *CBCT *cone beam computed tomography, *GDPR g*eneral data protection regulation

All included articles explicitly discussed and/or mentioned ethical considerations, including obtaining ethical approval, obtaining informed consent from participants and/or patients, and privacy [14–19]. Regarding legal awareness, both studies in the general dentistry and forensic fields raised concerns about the accuracy of using facial scan data [15], with all forensic studies implicitly mentioning the technical accuracy of the implementation system, particularly with respect to legal applications and surveillance contexts [17–19]. Additionally, regulatory aspects, such as medical device regulations (MDR) and broader medical regulatory considerations, were addressed in the studies published in 2025 [14, 16]. Purpose limitation and transparency were also mentioned as concerns [16].

## Discussion

This study represents the first facial scan review to broaden the scope to include the ethical and regulatory aspects of use in dentistry. Although facial scanning technologies have been in use since the early 2000s, their adoption in dental practice has outpaced awareness of the associated legal, regulatory, and ethical concerns. This gap is especially important because dentistry has seen the rapid use of commercial scanners, including smartphones, in private practices, often without institutional oversight or scientific validation. Our study highlights that despite the heavy discussion of biometrics elsewhere, this awareness has not translated to the dental field. The gap we are addressing is not merely a theoretical one, it is a practical, clinical vulnerability where practitioners are generating permanent biometric maps of patients without tailored regulatory guidance or sufficient ethical awareness, highlighting a practical and clinically relevant translational gap that has not yet been adequately addressed in dentistry.

The technical and certification review in the first part yielded limited data, with incomplete reporting of product standards and certifications. This deficit might occur because the scanner distributors, who may not be the actual manufacturers, do not provide the critical details. Technical information extends beyond quantifiable metrics used to compare scanner performance. The advancements directly shape regulatory and governance needs, as evolving facial scanning capabilities introduce new ethical and legal challenges that existing frameworks might not fully address. Therefore, technical information is essential not only for clinicians and researchers to assess clinical accuracy, but also for regulators to establish appropriate oversight mechanisms that ensure patient safety. Because many scanners identified might not first designed for medical purposes, there is a large gap between industrial and medical applications. Manufacturers need to consider the regulatory requirements for each field.

To the best of our knowledge and based on publicly available data, only one standalone facial scanner, aside from those integrated into CBCT units, was explicitly identified as MDR-certified. This compliance, marked by the scanners launched during the IDS 2025, was a good sign of a shift towards broader regulatory compliance. While the certification processes are undeniably complex, achieving compliance is essential, as it bridges the gap between raw technical capability and the professional trust required for patient safety. As for clinicians, inaccessible technical specifications, certification, and accreditation data are not an omission; they can create a chain of gaps that undermine the device’s validity. Without these specifications and declarations, clinicians cannot verify the device’s accuracy or compliance with safety standards relative to the benchmark. This lack of information could increase unquantifiable variables, leading to uncertainty in the diagnostic process and affecting the treatment plan. The dynamic nature of the market might also contribute to limited data availability, which likely explains some of the incomplete information identified in this review. The increased use of facial scans, driven by breakthrough technology in digital dentistry, might shift market demand and bypass institutional agencies that prioritize ethical and regulatory mandates, similar to what has happened with the use of artificial intelligence in the decision-making process [20, 21]. Although the commercialization of facial scanners is not as rapid as that of artificial intelligence-integrated software, the steady evolution of scanning technology and increase in use necessitate that clinicians should stay alert and up-to-date on the regulations. Specifically, the rise of cloud-based data processing and the storage of facial information could potentially introduce critical vulnerabilities. A clear definition of user liability, accountability, and robust protections for long-term data privacy to prevent unauthorized access or misuse are essential and should have been taken into account.

In general, both 2D and 3D facial images inherently contain detailed biometric information that can identify an individual. However, according to Katsanis et al. [22], participants expressed more concern about 3D facial images than 2D photographs in health research. Also, comfort with 3D facial images as biometrics was higher than with fingerprint data, but lower than with eye scans or DNA data [23]. In a clinical perspective, a 3D facial scan poses a greater risk of re-identification. As found by Steeg et al. [24], even colorless, anonymized magnetic resonance imaging head images can accurately identify individuals, posing a significant privacy risk and highlighting the need for data protection measures. In addition, it is the visual element of a virtual patient [25, 26]. While this model offers superior clinical utility for matching, follow-up, and surgical simulation, they also create a unique security paradox. Because a 3D scan is a permanent biometric map and highly identifiable, it cannot be reset or altered if it is leaked, leading to a permanent compromise of the patient’s identity. When handling this facial data, clinicians are recommended to obtain explicit consent and adhere to purpose limitation, ensuring the information is used only for its intended medical objective [27].

Technology trends were clearly observable. Technological advancement has progressed from early laser scanners to stereophotogrammetry and, more recently, to structured light systems. However, scientific validation and regulatory frameworks lag behind technological development, especially for smartphone-based applications, which show the lowest percentages among scanner types. Although smartphone solutions greatly expand accessibility, their accuracy is generally lower, and the use of inherently identifiable facial data introduces privacy risks. When downloading and using mobile applications, users or clinicians consent to data collection by developers through terms and conditions. This is problematic in clinical settings if patients are unaware of these data practices. Thus, clinicians must ensure that informed consent explicitly covers data processing and storage by any third-party software and that security safeguards are robust. Another important consideration is that the technological specifications reported in this study are based on publicly available information provided by manufacturers. Certain technical details, such as the use of hybrid or proprietary technologies, might not be fully disclosed due to confidentiality or commercial sensitivity. As a result, some aspects of scanner performance or design might remain inaccessible.

Based on this scoping review, nearly 500 human-based studies involving facial scanners were identified. However, only a small proportion explicitly mentioned and/or discussed ethical or regulatory considerations at any point in their manuscripts. This might be partly because most of the included studies were research-based, where ethical approval processes inherently cover consent and data protection through institutional review [28, 29]. Additionally, the inclusion criteria were primarily restricted to dentistry, which narrowed the scope of the included articles. Nevertheless, the majority of case reports and technical reports did not mention ethical approval, indicating that ethical safeguards can be overlooked, particularly in lower-level or demonstration-type studies. While the ethical and regulatory implications of facial imaging and biometric data have been extensively discussed in the broader medical and health-related literature, these considerations have received comparatively limited attention within the dental field. As facial scanners, smartphone-based facial imaging applications, and integrated digital workflows become increasingly adopted in dental practice and research, the ethical and regulatory challenges associated with the collection, storage, sharing, and analysis of facial data warrant greater consideration. This review addresses a discipline-specific gap by examining how ethical and regulatory issues are currently acknowledged and discussed in the dental facial imaging literature. The findings highlight the need for more awareness and consideration of ethical and regulatory issues within dental research and clinical practice. By identifying the current concerns in the current literature, this review provides a foundation and potential gaps for future discussions and may inform the development of field-specific recommendations and guidelines for the responsible use of facial imaging technologies in dentistry.

Among the identified ethical issues, informed consent and data privacy were the most frequently mentioned concerns, mentioned in 83% of the included studies [14–18]. These findings align with Tirapelli et al. [30], which reported that patients’ unawareness of how their data is processed or used is the main concern. This indicates the necessity of providing clear, purpose-specific information during the consent process. The introduction of major global data protection regulations, most prominently the European General Data Protection Regulation (GDPR) in 2016 [31], has reshaped and imposed stronger obligations on data use and processing. This created a butterfly effect in data privacy across the world, including the California Consumer Privacy Act and Illinois Biometric Information Privacy Act in the United States, Canadian Privacy Laws, Indian Data Protection Bill, Chinese Personal Information Protection Law, Singapore Personal Data Protection Act, Japan’s Act on Protection of Personal Information, and Australian Privacy Act [32, 33]. As these GDPR-like regulations become stricter and are adopted worldwide, the old status quo of unregulated data is vanishing. Compliance is no longer a choice, but a global prerequisite. As data privacy legislations diverge across countries, practitioners in an increasingly mobile society must be careful about cross-border data transfer to ensure seamless patient care.

Technological accuracy was identified as a concern in all included forensic studies [16–18], particularly in facial recognition applications. In these contexts, even minor errors in identification can lead to several legal consequences, such as wrongful accusation or compromise. For instance, a false-positive match in a forensic investigation can result in the detention of an innocent individual or in incomplete evidence due to insufficient accuracy or reliability [34, 35]. Limited technical information provided leaves researchers and clinicians unable to assess the tool’s suitability and reliability. While regulatory frameworks and certification mandates require manufacturers to disclose safety standards, a transparency gap remains. This potential lack of oversight might create clinical uncertainty, forcing practitioners to rely on unverified data and ultimately compromising the precision of diagnosis and patient care. Although facial scanners are non-invasive and pose no physical risk, a clear and robust ethical and regulatory framework is necessary, as facial scan data is unique, identifiable, and cannot be fully anonymized. Establishing such a framework is essential to guarantee professional integrity, provide legal certainty, and, most importantly, safeguard the patient’s fundamental rights.

Overall, the selection and use of facial scanners in dentistry must balance technical performance with ethical and regulatory responsibilities shared by both manufacturers and clinicians. In larger hospitals or clinical centers, scanner selection may also be guided by institutional committee decisions, which can introduce another layer of regulatory oversight or potential bias. Core ethical principles and code of professional conduct, as outlined by the American Dental Association (ADA) and the Federation of European Dental Competent Authorities and Regulators (FEDCAR), including patient autonomy, non-maleficence, beneficence, justice, and veracity, should be taken into account. In addition, the Council of European Dentists clearly states in its purpose and guiding principles that practitioners should apply the current standards of practice. Dentists must also comply with national law and ethical customs governing the practice of the profession, and must operate in compliance with EU and national legislation, as well as the applicable professional code of conduct. Although facial scanning technologies have not yet been explicitly or consistently addressed as a distinct data type in existing professional guidelines, the findings of this study indicate that the facial scanning technologies and applications require a framework that aligns with these established ethical and professional standards. In the absence of formal guidance specific to facial scan use in both medicine and dentistry, a multidisciplinary collaboration between clinical, research, legal, technological industry, and patient stakeholders is essential to develop a standardized consensus framework that could help clinicians maintain compliance, mitigate risk, and uphold high standards of professional practice.

A primary limitation of this review is its focus on dentistry, which may overlook broader medical or engineering perspectives. The limited information available on official websites, combined with low response rates from companies and manufacturers, further hindered the completeness of the collected data. The rapidly evolving facial-scanning market presents methodological challenges for comprehensive technology assessments [36]. Although information from manufacturer websites and publicly available sources may change over time, this approach was adopted to capture devices currently available in clinical practice, many of which have not yet been evaluated in scientific validation studies. However, similar methodologies have been used in previous overviews of emerging imaging technologies [37]. It should be noted that the information gathered through this process reflects manufacturer-reported and publicly available marketing material rather than independently verified regulatory or certification status. As such, reported accreditation and certification details should be interpreted as claims made by manufacturers rather than confirmed regulatory approval, and future studies may benefit from independent verification through official regulatory databases. The findings should be interpreted with caution, as some information could not be independently verified, and manufacturer responses were not always available. Importantly, an unverifiable accreditation status does not indicate the absence of certification; rather, it reflects that certification information could not be identified or verified through the predefined data collection process, particularly for discontinued products or devices with limited publicly accessible documentation. The initial rationale for assessing company websites was to simulate the actual purchasing environment of clinicians, thereby highlighting the limited transparency manufacturers provide regarding clinical validation and regulatory status. However, reliance on website information alone is insufficient for a definitive regulatory audit, as such data may be incomplete or marketing-oriented. Nevertheless, the observed gaps underscore a critical issue: clinicians are expected to make informed decisions in an environment where essential regulatory and safety information is not readily accessible. This reinforces the need for more standardized, transparent reporting requirements for devices marketed and publicly available database for healthcare professionals. Nonetheless, this work highlights essential gaps in ethical and regulatory practice and provides a foundation for future development of responsible guidelines for facial scan use in dentistry. Conducting information updates every 5 years is recommended to capture significant trends and the rapid pace of advancement in this sector.

## Conclusion

Despite evidence supporting the clinical validity of facial-scanning technologies, only a limited proportion of identified scanners had publicly accessible, verifiable certification under the Medical Device Regulation, and ethical and regulatory aspects were underrepresented in the published literature. These findings should not be interpreted as evidence that such considerations are absent from clinical practice, but rather that they are often insufficiently documented in scientific literature. The topic, including informed consent, data privacy, accuracy, and medical device compliance, was addressed in the included studies. More transparency in reporting, clearer consideration of regulatory status, and the development of dentistry-specific recommendations may support the responsible integration of facial-scanning technologies in both research and clinical translation.

## Supplementary information

Below is the link to the electronic supplementary material.

Supplementary Material 1

## Data Availability

Data related to technical specifications and certification status have been deposited in a publicly accessible web archive ( [https://archive.org/details/@thanatchaporn\_jindanil/web-archive](https:/archive.org/details/@thanatchaporn_jindanil/web-archive) ). Additional data supporting the findings of this study are available from the corresponding author upon reasonable request.

## References

[1] Antonacci D, Caponio VCA, Troiano G et al (2023) Facial scanning technologies in the era of digital workflow: A systematic review and network meta-analysis. J Prosthodont Res 67:321–336. 10.2186/jpr.JPR_D_22_00107 36058870

[2] Lee GH, Park JH, Park JJ et al (2024) Key considerations for efficient 3-dimensional data integration with a face scanner in the digital era: Product review of the hybrid face scanner. AJO-DO Clin Companion 4:351–359. 10.1016/j.xaor.2024.06.004

[3] Singh P, Bornstein MM, Hsung RT et al (2024) Frontiers in Three-Dimensional Surface Imaging Systems for 3D Face Acquisition in Craniofacial Research and Practice: An Updated Literature Review. Diagnostics (Basel) 14:423. 10.3390/diagnostics14040423 PMC10888365 38396462

[4] Kovacs L, Zimmermann A, Brockmann G et al (2006) Accuracy and precision of the three-dimensional assessment of the facial surface using a 3-D laser scanner. IEEE Trans Med Imaging 25:742–754. 10.1109/TMI.2006.873624 16768239

[5] Gibelli D, Dolci C, Cappella A et al (2020) Reliability of optical devices for three-dimensional facial anatomy description: a systematic review and meta-analysis. Int J Oral Maxillofac Surg 49:1092–1106. 10.1016/j.ijom.2019.10.019 31786104

[6] Quinzi V, Polizzi A, Ronsivalle V et al (2022) Facial Scanning Accuracy with Stereophotogrammetry and Smartphone Technology in Children: A Systematic Review. Child (Basel) 9:1390. 10.3390/children9091390 PMC9498045 36138698

[7] Jindanil T, Xu L, Fontenele RC et al (2024) Smartphone applications for facial scanning: A technical and scoping review. Orthod Craniofac Res 27(Suppl 2):65–87. 10.1111/ocr.12821 PMC11654360 38842250

[8] Piedra-Cascón W, Meyer MJ, Methani MM, Revilla-León M (2020) Accuracy (trueness and precision) of a dual-structured light facial scanner and interexaminer reliability. J Prosthet Dent 124:567–574. 10.1016/j.prosdent.2019.10.010 31918895

[9] Ntovas P, Revilla-León M, Barmak AB et al (2025) Accuracy of 3-dimensional virtual patient representation using different digital integration techniques and four facial scanners: A clinical validation study. J Prosthet Dent S 0022–3913:00838–00838. 10.1016/j.prosdent.2025.10.029 41173712

[10] Qandeel M (2024) Facial recognition technology: regulations, rights and the rule of law. Front Big Data 7:1354659. 10.3389/fdata.2024.1354659 PMC11183273 38895177

[11] European Data Protection Board (2022) Guidelines 05/2022 on the use of facial recognition technology in the Area of Law Enforcement

[12] Kováč P, Jackuliak P, Bražinová A et al (2024) Artificial Intelligence-Driven Facial Image Analysis for the Early Detection of Rare Diseases: Legal, Ethical, Forensic, and Cybersecurity Considerations. AI 5:990–1010. 10.3390/ai5030049

[13] Rokhshad R, Karteva T, Chaurasia A et al (2024) Artificial intelligence and smile design: An e-Delphi consensus statement of ethical challenges. J Prosthodont 33:730–735. 10.1111/jopr.13858 38655727

[14] Darbari Kaul R, Duong C, Ma J et al (2025) A comparison of the accuracy and feasibility of a low-cost mobile application versus higher-cost handheld 3D scanner for digital ear prosthetics. ANZ J Surg 95:719–726. 10.1111/ans.19374 39731345

[15] Jindanil T, Burlacu-Vatamanu OE, Meyns J et al (2024) Automated orofacial virtual patient creation: A proof of concept. J Dent 150:105387. 10.1016/j.jdent.2024.105387 39362299

[16] Jindanil T, Gracea RS, Claessens N et al (2025) Three-dimensional facial scanning: Quantitative and qualitative assessment of five different modalities with CBCT as a reference. J Dent 163:106179. 10.1016/j.jdent.2025.106179 41106459

[17] Koudelová J, Dupej J, Brůžek J et al (2015) Modelling of facial growth in Czech children based on longitudinal data: Age progression from 12 to 15 years using 3D surface models. Forensic Sci Int 248:33–40. 10.1016/j.forsciint.2014.12.005 25576677

[18] Koudelová J, Hoffmannová E, Dupej J, Velemínská J (2019) Simulation of facial growth based on longitudinal data: Age progression and age regression between 7 and 17 years of age using 3D surface data. PLoS ONE 14:e0212618. 10.1371/journal.pone.0212618 PMC6386244 30794623

[19] Lynnerup N, Clausen ML, Kristoffersen AM, Steglich-Arnholm H (2009) Facial recognition and laser surface scan: A pilot study. Forensic Sci Med Pathol 5:167–173 10.1007/s12024-009-9094-8 19507073

[20] Winecoff AA, Watkins EA (2022) Artificial concepts of artificial intelligence: institutional compliance and resistance in AI startups. Proceedings of the 2022 AAAI/ACM conference on AI, ethics, and society. Association for computing machinery: Oxford, United Kingdom 788–799. 10.1145/3514094.3534138

[21] Naik N, Hameed B, Shetty D et al (2022) Legal and Ethical Consideration in Artificial Intelligence in Healthcare: Who Takes Responsibility? Front Surg 9:862322. 10.3389/fsurg.2022.862322 PMC8963864 35360424

[22] Katsanis SH et al (2021) A survey of U.S. public perspectives on facial recognition technology and facial imaging data practices in health and research contexts. PLoS ONE 16:e0257923. 10.1371/journal.pone.0261738 PMC8516205 34648520

[23] Katsanis SH, Claes P, Doerr M et al (2022) U.S. Adult Perspectives on Facial Images, DNA, and Other Biometrics. IEEE Trans Technol Soc 3:9–15. 10.1109/tts.2021.3120317 PMC8965792 35360665

[24] Steeg K, Bohrer E, Schäfer SB et al (2024) Re-identification of anonymised MRI head images with publicly available software: investigation of the current risk to patient privacy. eClinicalMedicine 78:102930. 10.1109/tts.2021.3120317 PMC11617779 39640939

[25] Haku-Mizuhara K, Tanabe G, Kadempour A, Mirafzali S, Yotsuya M, Yamaguchi S (2026) Development of a 4-dimensional virtual patient in motion: A digital concept. J Prosthet Dent 135:47–53. 10.1016/j.prosdent.2025.02.064 40169343

[26] Amarnath G, Govula K, Sannapureddy S et al (2025) Digital Twins in Dentistry: A Narrative Review of Current Applications and Future Horizons. J Int Oral Health 17:447–452. 10.4103/jioh.jioh_230_25

[27] Bennett KG, Bonawitz SC, Vercler CJ (2019) Guidelines for the Ethical Publication of Facial Photographs and Review of the Literature. Cleft Palate Craniofac J 56:7–14. 10.1177/1055665618774026 29715061

[28] European Commission (2021) Informed consent to data processing. Ethics and data protection 11–14

[29] Mörch CM, Atsu S, Cai W et al (2021) Artificial Intelligence and Ethics in Dentistry: A Scoping Review. J Dent Res 100:1452–1460. 10.1177/00220345211013808 34060359

[30] Tirapelli C, Gaêta-Araujo H, Costa ED et al (2025) Patient perceptions of artificial intelligence in dental imaging diagnostics: a multicentre survey. Dentomaxillofac Radiol 54:427–436. 10.1093/dmfr/twaf018 40080713

[31] The European Parliament and the Council of the European Union. Regulation (EU) 2016/679 (General Data Protection Regulation) (2016) Official Journal of the European Union

[32] Simmons D, GDPR-like Data Privacy Laws (2022) 17 Countries with. Update Jan 13, 2022 (accessed on 2026 6/1). Available from: https://insights.comforte.com/countries-with-gdpr-like-data-privacy-laws. Accessed 14 Jan 2026

[33] Wen Y, Liu B, Song L et al (2024) Facial recognition technology and the privacy risks, in face De-identification: Safeguarding identities in the digital era. Springer, Cham 15–20. 10.1007/978-3-031-58222-6_2

[34] National Institute of Justice (2023) The impact of false or misleading forensic evidence on wrongful convictions. National Institute of Justice

[35] Morgan J (2023) Wrongful convictions and claims of false or misleading forensic evidence. J Forensic Sci 68:908–961. 10.1111/1556-4029.15233 36946413

[36] Thurzo A, Varga I (2025) Revisiting the Role of Review Articles in the Age of AI-Agents: Integrating AI-Reasoning and AI-Synthesis Reshaping the Future of Scientific Publishing. Bratisl Med J 126:381–339. 10.1007/s44411-025-00106-8

[37] Gaêta-Araujo H, Alzoubi T, Vasconcelos KF et al (2020) Cone beam computed tomography in dentomaxillofacial radiology: a two-decade overview. Dentomaxillofac Radiol 49:20200145. 10.1259/dmfr.20200145 PMC7719864 32501720

